# Fatigue in Hemodialysis Patients: A Comparative Analysis with Healthy Controls

**DOI:** 10.3390/ejihpe15020012

**Published:** 2025-01-26

**Authors:** Leszek Sułkowski, Andrzej Matyja, Maciej Matyja

**Affiliations:** 1 Department of General Surgery, Regional Specialist Hospital, 42-218 Czestochowa, Poland; leszeksulkowski@szpitalparkitka.com.pl; 2 2nd Department of General Surgery, Jagiellonian University Medical College, 30-688 Krakow, Poland

**Keywords:** fatigue, quality of life, hemodialysis, end-stage renal disease, chronic kidney disease, Modified Fatigue Impact Scale (MFIS), Modified Fatigue Impact Scale-5-item version (MFIS-5), WHOQOL-BREF

## Abstract

This study investigates fatigue and quality of life in hemodialysis patients, examining the influence of demographic and clinical factors on these outcomes. A cohort of 115 hemodialysis patients and 112 healthy controls completed the Modified Fatigue Impact Scale (MFIS), the shorter MFIS-5, and the WHOQOL-BREF quality of life assessment. The findings indicate that hemodialysis patients experience significantly higher levels of fatigue, which correspond with lower quality of life, particularly in the physical and psychological domains, compared to healthy controls. Male patients reported significantly higher levels of fatigue and lower quality of life scores, whereas younger patients demonstrated relatively better outcomes. Extended dialysis sessions exceeding four hours were associated with poorer social well-being, and educational attainment was positively linked with physical and environmental quality of life domains. However, marital status did not show a significant effect. The study validates the consistency between MFIS and MFIS-5 scores, recommending MFIS-5 for time-sensitive clinical use without compromising accuracy. These results underscore the need for individualized, multi-dimensional approaches to fatigue management in hemodialysis patients, emphasizing interventions that address physical, psychological, and social well-being to enhance overall quality of life. The findings highlight specific factors that may guide tailored support strategies to improve patient outcomes in this population.

## 1. Introduction

End-stage renal disease necessitating hemodialysis is a prevalent chronic condition with increasing incidence (Horigan, 2012) Patients receiving in-center dialysis face numerous physiological and psychological symptoms, requiring significant lifestyle adjustments and compliance with medical demands (Sousa et al., 2019; Ju et al., 2018; Flythe et al., 2019). Chronic kidney disease and dialysis impact metabolic balance and disrupt body image and quality of life, likely contributing to fatigue (Zazzeroni et al., 2017; Meca-Lallana et al., 2019; Tsirigotis et al., 2022). Fatigue is one of the most commonly reported symptoms among hemodialysis patients, affecting between 60% and 97% of this population (Horigan, 2012; Flythe et al., 2019). This symptom negatively impacts both physical and mental functioning and is associated with increased mortality risk, depression, and decreased quality of life, as well as with cardiac events (Horigan, 2012; Flythe et al., 2019). Additionally, depression is widely observed in this patient population and is closely linked with insomnia and fatigue (Sakiqi et al., 2022). According to Sakiqi et al., elevated levels of fatigue correlate with a higher incidence of depression (Sakiqi et al., 2022). Persistent fatigue is common among hemodialysis patients, primarily due to chronic kidney disease, and is often worsened following dialysis sessions (Ju et al., 2018; Horigan & Barroso, 2016). As a result, a standard recommendation for these patients is to rest for approximately five hours following each dialysis session (Horigan & Barroso, 2016). Fatigue severely impacts physical and mental functionality, activity levels, and quality of life, presenting a significant burden for patients, their families, and healthcare providers (Horigan, 2012; Flythe et al., 2019; Horigan & Barroso, 2016; Bossola et al., 2017; Wolfgram, 2019).

The challenges of life on hemodialysis go beyond achieving optimal mineral balance and Kt/V ratios. While dialysis parameters such as Kt/V, urea reduction ratio (URR), and ultrafiltration are critical for healthcare providers in assessing treatment efficacy and predicting prognosis, patients often place minimal emphasis on these values. Instead, they focus on quality of life and the degree to which dialysis interferes with their day-to-day activities (Pojatić et al., 2022; Edwards & Manera, 2022). Hemodialysis requires patients to dedicate several hours per session, three times weekly, which significantly affects both personal and professional life (Zazzeroni et al., 2017; Jordakieva et al., 2020). Consequently, care should focus on supporting patients’ sense of well-being and enabling them to engage in meaningful activities (Edwards & Manera, 2022). Quality of life has become a primary outcome metric in assessing hemodialysis treatment efficacy (Zazzeroni et al., 2017). Studies indicate that the quality of life in chronic kidney disease patients decreases progressively with the disease stage, highlighting its importance as an outcome metric (Kefale et al., 2019). Therefore, it is crucial for healthcare providers to design treatments not only to meet optimal biochemical and laboratory benchmarks but also to enhance quality of life and alleviate symptoms, such as fatigue (Horigan, 2012; Zazzeroni et al., 2017; Sułkowski et al., 2023; Kušleikaitė et al., 2010; Gembillo et al., 2021). Providers must continually evaluate whether patients are achieving their personal health goals. Each patient should receive personalized, patient-centered care and play an active role in their treatment (Edwards & Manera, 2022).

The physical and mental quality of life of hemodialysis patients is inherently limited by the treatment itself, which is linked to increased mortality risk (Zazzeroni et al., 2017; Kraus et al., 2016). Physical quality of life, already compromised by chronic kidney disease, is further impaired by reduced mobility and health complications (Kraus et al., 2016). Similar findings have been reported in studies ranking diseases by their impact on quality of life, where chronic kidney diseases were found to lower physical and mental health dimensions substantially (Krawczyk-Suszek et al., 2024). Mental quality of life is affected by depressive symptoms, social isolation, anxiety, and a lack of positive affect (Kraus et al., 2016). Furthermore, chronic diseases have been identified as conditions significantly lowering health-related quality of life, particularly in physical and mental health domains (Krawczyk-Suszek et al., 2024). Implementing strategies to manage fatigue has shown to improve the quality of life in this patient group (Horigan, 2012).

Although fatigue is well documented among hemodialysis patients, few studies quantify the specific impact of hemodialysis and demographic factors on fatigue and quality of life (Sułkowski et al., 2025). Therefore, this study aims to investigate the influence of fatigue on hemodialysis patients, identify particularly vulnerable subgroups, and assess the perceived quality of life among participants. This study explores the impact of demographic and dialysis-related factors on quality of life, as defined by the World Health Organization (WHO) as a state of complete physical, mental, and social well-being (Szabo, 1996; The WHOQOL Group, 1998b). To this end, the Modified Fatigue Impact Scale (MFIS) and its abbreviated five-item version (MFIS-5) were used to measure fatigue, while quality of life was assessed using the WHO Quality of Life questionnaire (WHOQOL-BREF). Additionally, the study examines whether the full MFIS aligns with the shortened MFIS-5, thereby evaluating the utility of the shortened version in clinical settings for hemodialysis patients.

## 2. Materials and Methods

With authorization from the Bioethics Committee (K.B.Cz.0014/2017), we conducted an in-depth evaluation of the quality of life and life satisfaction among a cohort of dialysis patients, examining the effect of dialysis treatment on various life aspects, such as physical fatigue, social support, vision, and sexual dysfunction (Sułkowski et al., 2023; Sułkowski et al., 2018; Sułkowski et al., 2024).

Participants were recruited from a single Dialysis Unit in a Regional Specialist Hospital. Inclusion criteria included: male and female patients aged ≥18 years, diagnosed with end-stage renal disease, receiving hemodialysis three times per week, and providing consent to participate in this study. Exclusion criteria were age below 18, acute kidney failure, refusal to participate, and incomplete questionnaire responses. All participants provided written informed consent and completed a demographic survey collecting data on sex, age, education, and marital status [Table 1]. This information was supplemented with medical records detailing hemodialysis session duration, Kt/V, type of vascular access (arteriovenous fistula vs. central venous catheter), and URR [Table 2]. Associations between demographic characteristics, medical parameters, and questionnaire subscales were also examined. The control group comprised individuals without kidney disease or other chronic conditions requiring specialized treatment. Both hemodialysis patients and controls completed the MFIS, its abbreviated 5-item version MFIS-5, and WHOQOL-BREF questionnaires, with scoring ranges and subscales outlined in Table 3.

MFIS and its shortened 5-item version, MFIS-5, are established tools for assessing fatigue in both clinical and research contexts across various patient groups (D’Souza, 2016). The MFIS questionnaire comprises 21 items from the Fatigue Impact Scale, organized into physical, cognitive, and psychosocial subscales, each contributing to an overall fatigue score (D’Souza, 2016). MFIS-5 is a concise version of the MFIS, containing five items that most strongly correlate with the total MFIS score: two from the cognitive subscale, two from the physical subscale, and one from the psychosocial subscale (Meca-Lallana et al., 2019; D’Souza, 2016; Cozart et al., 2021). Participants select one response per question—ranging from “almost always” to “never”—to describe how fatigue affected them in the past four weeks. Responses are scored from 0 to 4, producing a total score of 0 to 20 points for MFIS-5 and 0 to 84 points for MFIS. Higher scores indicate greater levels of fatigue (D’Souza, 2016).

The WHOQOL-BREF questionnaire assesses individuals’ perceived quality of life, independent of disease or disability status. It consists of 26 items divided into four domains: physical health, psychological health, social relationships, and environment (Szabo, 1996; The WHOQOL Group, 1998b; The WHOQOL Group, 1998a; The WHOQOL Group, 1998b; The WHOQOL Group, 1998a; Skevington et al., 2004). The physical health domain includes questions on daily activities, dependence on medicinal aids, energy and fatigue, mobility, pain and discomfort, work capacity, sleep, and rest. The psychological domain evaluates body image, positive and negative feelings, self-esteem, spirituality, religion, personal beliefs, thinking, learning, memory, and concentration. The social relationships domain addresses personal relationships, social support, and sexual activity, while the environment domain considers financial resources, physical safety and security, healthcare access, home environment, opportunities for learning, physical surroundings, and leisure activities. Higher scores in these domains reflect a better quality of life (Szabo, 1996; The WHOQOL Group, 1998b; Sułkowski et al., 2018; The WHOQOL Group, 1998a; The WHOQOL Group, 1998b; The WHOQOL Group, 1998a; Skevington et al., 2004).

### Statistical Analysis

Frequency, range, and mean values were calculated for demographic data. Standard deviations (SD) were computed for continuous variables. To compare variables, the Student’s *t*-test was employed, with statistical significance defined as *p* < 0.05. Prior to conducting the Student’s *t*-tests, the Shapiro–Wilk test was performed to evaluate the normality of the data distribution for each group. The results indicated that the data met the assumption of normality necessary for parametric testing. The statistical analyses did not include adjustments for multiple comparisons, which may increase the risk of Type I error. All analyses were performed using IBM SPSS Statistics (Version 27.0, IBM Corp., Armonk, NY, USA).

## 3. Results

Of the 136 initially screened hemodialysis patients, 115 met the inclusion criteria, with a median age of 63.7 years (SD = 11.5) and 79 males (68.7%) [Table 3]. The control group included 112 employees of the Dialysis Unit and their adult relatives, with a median age of 60.6 years (SD = 11.9) and 76 males (67.9%) [Table 3].

The present study found that hemodialysis patients scored significantly lower in the physical and psychological health domains of the WHOQOL-BREF (*p* < 0.0001 for each) and significantly higher on the physical and psychosocial subscales of the MFIS, as well as on the total MFIS and the abbreviated MFIS-5 scores (*p* < 0.0001 for each) [Table 3].

Table 1 presents the associations between demographic factors (sex, age, education, and marital status) and both the MFIS and WHOQOL-BREF questionnaire scores. Male hemodialysis patients scored lower in the physical, psychosocial health, and environment domains of the WHOQOL-BREF and reported greater levels of fatigue, with higher scores on each MFIS subscale, including the MFIS-5. However, these differences were not statistically significant when comparing dialyzed females to healthy females [Table 1]. To assess the influence of age on test scores, a median age of 65 years was established. No statistically significant differences were found in WHOQOL-BREF scores among dialysis patients based on age; however, younger hemodialysis patients (below the median age) reported less fatigue, reflected by lower scores on each MFIS subscale, total MFIS, and MFIS-5 questionnaires [Table 1]. In terms of education level, statistically significant differences were found in the physical health and environment domains of the WHOQOL-BREF, while no significant effect of education was observed on the MFIS. Additionally, marital status did not influence the scores on either the MFIS or WHOQOL-BREF questionnaires [Table 1].

We also evaluated the influence of dialysis-related factors, such as hemodialysis session duration, Kt/V, vascular access modality, and URR, on questionnaire scores [Table 2]. The findings demonstrated a mean hemodialysis session duration of 4 h. Patients with session durations exceeding four hours (n = 22) reported lower scores on the MFIS psychosocial subscale and the WHOQOL-BREF social relationship domain, reflecting greater impairments in social and psychosocial well-being [Table 2]. No statistically significant differences were observed between patients with Kt/V values below versus above the median across any of the questionnaires, including their subscales and MFIS-5 scoring. Furthermore, no significant differences were found based on vascular access type, with arteriovenous fistula and central venous catheter groups showing comparable scores across the analyzed questionnaires. Similarly, no significant distinctions were identified in any questionnaire scores when comparing patients with URR values below and above the median [Table 2].

## 4. Discussion

Both physical and mental fatigue are among the most frequently reported symptoms in hemodialysis patients, severely impacting daily functioning and overall quality of life (Horigan & Barroso, 2016; Bossola et al., 2017). A considerable proportion of individuals receiving in-center hemodialysis, both adults and minors, report experiencing fatigue (Flythe et al., 2019; Theofilou, 2011; Karava et al., 2022). Our findings indicate that hemodialysis patients are more prone to fatigue than healthy individuals, as assessed through the MFIS and MFIS-5 questionnaires [Table 3]. Hemodialysis patients are required to adhere to several lifestyle modifications and clinical guidelines (Sousa et al., 2019). Both objective and subjective factors are proposed to influence fatigue severity in this population (Ju et al., 2018). Furthermore, our analyses revealed that hemodialysis patients scored lower on the physical and psychosocial subscales of the MFIS, and lower on physical and psychological health domains of the WHOQOL-BREF [Table 3]. Impaired physical health, limited mobility, and dissatisfaction with physical ability collectively reduce physical quality of life in this cohort (Kraus et al., 2016). Social support from family, significant others, and healthcare professionals is associated with better treatment outcomes in chronic illness (Sousa et al., 2019). Karadag et al. reported a significant negative correlation between fatigue severity and social support from family, friends, significant others, and overall support, suggesting that greater support can reduce fatigue levels (Karadag et al., 2013).

Higher MFIS physical health scores reported by patients are consistent with lower WHOQOL-BREF physical health scores. The WHOQOL-BREF physical health domain assesses daily activities, dependence on medical aids, energy, fatigue, mobility, pain, work capacity, sleep, and rest (The WHOQOL Group, 1998b; The WHOQOL Group, 1998a). These findings may relate to the chronic fatigue experienced by these patients, including post-dialysis fatigue, which worsens following each session. Horigan and Barroso identified two fatigue patterns in hemodialysis: continuous fatigue and post-dialysis fatigue (Horigan & Barroso, 2016). Post-dialysis fatigue often eludes clinical markers such as dialysis adequacy, nutrition, or activity (Horigan & Barroso, 2016). Studies have shown that most hemodialysis patients experience a drop in blood pressure during dialysis, with about a third experiencing hypotension (Wolfgram, 2019). Consequently, patients require additional rest—up to five hours post-dialysis—due to this fatigue (Horigan & Barroso, 2016). Our study also found poorer scores on the MFIS psychosocial scale and the WHOQOL-BREF psychological health domain. The psychological health domain evaluates factors such as body image, self-esteem, emotions (both positive and negative), spirituality, personal beliefs, and cognitive functions (The WHOQOL Group, 1998b; The WHOQOL Group, 1998a). The literature suggests that prolonged dialysis sessions and extended time in dialysis units reduce social interaction and exacerbate physical limitations, diminishing opportunities for fulfilling activities. Social support, spirituality, and participation in support networks contribute to reduced depressive symptoms in this population (Gerogianni et al., 2019).

Interestingly, there were no statistically significant differences between dialysis patients and healthy controls in the social relationship and environment domains of the WHOQOL-BREF. These domains cover personal relationships, social support, physical safety, and access to resources. It should be noted that the healthy controls in this study were employees of the Dialysis Unit and their relatives, making this a unique group. The shared professional environment between patients and caregivers may influence these results, as caregivers and patients both face time away from home. Additionally, no statistically significant differences were observed in the MFIS cognitive subscale. Most of the control group females were dialysis unit nurses, likely influencing outcomes since they spent considerable time in a high-stress environment (Sułkowski et al., 2024). Future research is necessary to explore cognitive aspects more thoroughly.

The impact of demographic factors, including age, sex, education level, and marital status, on fatigue and quality of life in hemodialysis patients has been examined. Our findings reveal differing impacts of hemodialysis on males and females. Males undergoing dialysis reported significantly higher MFIS and MFIS-5 scores, with lower scores in physical, cognitive, and psychosocial subscales of the MFIS and worse quality of life on the WHOQOL-BREF domains of physical, psychological, and environmental health, compared to healthy males. No statistically significant differences were observed in MFIS or WHOQOL-BREF scores between female dialysis patients and healthy females. This gender disparity requires further investigation. The elevated fatigue reported by males may be attributed to disease severity. The lower self-reported quality of life in physical, psychosocial, and environmental domains in dialyzed males likely reflects the physical burden of chronic kidney disease (Kušleikaitė et al., 2010; Theofilou, 2011; Apostolou, 2007). Our control group included dialysis unit employees and their relatives, primarily female nurses working long hours in shifts, which may have affected the study outcomes. It is plausible that dialysis patients and caregivers experience similar fatigue levels; however, studies comparing patient and caregiver fatigue are limited.

Age is a consistent predictor of quality of life among hemodialysis patients. Research by Apostolou revealed poorer physical functioning in older hemodialysis patients, although social and mental health scores were comparable to those of younger patients (Apostolou, 2007). Our study identified lower MFIS-5 and MFIS scores in older patients across all subscales. This correlation may be due to age-related factors, such as muscle loss, poor sleep quality, and comorbidities, which often accompany fatigue (Tsirigotis et al., 2022; Sakiqi et al., 2022). While not explicitly evaluated in this study, comorbidities are known to influence fatigue and depression in aging patients (Tsirigotis et al., 2022). Tsirigotis et al. documented a strong association between fatigue and variables such as age, education level, comorbidities, and insomnia in hemodialysis patients, which clinicians may misattribute to age or dialysis side effects (Tsirigotis et al., 2022). WHOQOL-BREF results showed similar quality of life scores in both age groups, suggesting that, despite greater fatigue, older patients’ overall quality of life remains comparable to that of younger hemodialysis patients.

Our findings indicate that educational level does not influence fatigue in hemodialysis patients; however, higher education correlates with improved quality of life in physical and environmental domains. These findings align with prior research showing that high income and higher educational attainment are associated with improved physical and mental QoL, likely due to better access to healthcare and resources (Kefale et al., 2019). Reduced physical quality of life in hemodialysis patients is often marked by decreased mobility, dissatisfaction with physical capacity, and physical health limitations (Kraus et al., 2016). This association likely reflects the broader socioeconomic benefits linked to higher education. Furthermore, our results showed no significant association between marital status and fatigue or quality of life in any of the WHOQOL-BREF domains, suggesting that additional attention should be given to patients with lower educational attainment who may underreport physical and environmental quality of life.

Hemodialysis patients commonly report fatigue as a key symptom that impacts work-life balance, thereby diminishing overall quality of life (Meca-Lallana et al., 2019; Tsirigotis et al., 2022). To assess the role of dialysis parameters on fatigue and quality of life, we examined Kt/V, an indicator of dialysis efficacy. Our findings align with prior studies, confirming no correlation between Kt/V and fatigue levels in hemodialysis patients (Horigan, 2012). Additional analyses of dialysis session duration, type of vascular access, and URR showed no statistically significant associations with fatigue levels or physical and cognitive dimensions of fatigue. Only the psychosocial aspect of fatigue, as measured by the MFIS, was negatively impacted by longer dialysis sessions. Patients undergoing sessions longer than 4 h scored lower on the WHOQOL-BREF social relationships domain, potentially due to reduced social interactions and leisure time. Our findings suggest that, among dialysis-related factors, only session duration significantly affects the psychosocial dimension of fatigue and social relationships. Therefore, healthcare providers should consider session length as a factor influencing hemodialysis patients’ quality of life.

Our results from the MFIS indicate that similar results were obtained for both the full and abbreviated MFIS-5 scores. While the full MFIS is generally recommended for its comprehensive subscales (Meca-Lallana et al., 2019; D’Souza, 2016; Cozart et al., 2021), the MFIS-5 offers a practical alternative in time-sensitive clinical settings. Our findings support the MFIS-5 as a useful, user-friendly tool for quantifying fatigue among hemodialysis patients, though its limitations should be considered.

### Limitations

Our study has several limitations. Primarily, it is a single-site study involving only adult participants, which may limit the generalizability of the findings. Additionally, the selection of the healthy control group from among dialysis unit employees and their relatives may have influenced the results. In this control group, most females were nurses working in the dialysis unit, while the majority of males were relatives of these nurses. This composition may affect the outcomes, as nurses in the dialysis unit are subject to occupational stress and fatigue, which could potentially influence their responses in comparison to non-medical participants.

However, the selection of this control group also provided a unique perspective, enabling us to observe and compare fatigue levels between hemodialysis patients and the nurses caring for them. This comparison underscores the need to consider the well-being of healthcare workers in dialysis settings, as their fatigue levels may resemble those experienced by patients.

Despite these limitations, which suggest the need for further research with a broader and more diverse sample, our study offers valuable insights into the fatigue experienced by the hemodialysis patient population. This information can contribute to the development of targeted interventions aimed at improving patient quality of life and managing fatigue more effectively in clinical practice.

## 5. Conclusions

This study observed that individuals undergoing hemodialysis experienced higher levels of fatigue compared to healthy individuals. Furthermore, the effects of fatigue and quality of life outcomes appeared to differ between males and females. Specifically, male hemodialysis patients reported higher levels of fatigue across all aspects, alongside lower scores in social support, physical health, environmental, and psychological health domains. Conversely, female hemodialysis patients reported greater social support but lower retrospective memory scores.

The study also identified a negative correlation between age and fatigue levels among hemodialysis patients. Furthermore, university graduates undergoing hemodialysis reported better physical health and environmental scores. Extended hemodialysis sessions notably influenced psychosocial aspects of life. It is also essential to consider the fatigue levels of dialysis nurses, which may be comparable to those of dialysis patients.

Our findings suggest that the scores of the full MFIS and the abbreviated MFIS-5 were comparable, allowing the recommendation of the 5-item MFIS-5 version in situations where time constraints exist. However, the full version offers the added benefit of a detailed breakdown into subscales.

The insights provided by this study contribute to a deeper understanding of fatigue and quality of life in hemodialysis patients. Healthcare providers can use these findings to implement strategies to mitigate the effects of fatigue. The judicious use of MFIS and WHOQOL-BREF assessments can aid in risk stratification, facilitating early diagnosis and intervention for fatigue. Moreover, healthcare providers should actively educate patients about fatigue symptoms and offer support to help manage fatigue, thereby improving overall quality of life and well-being.

## Figures and Tables

**Table 1:** Demographic factors affecting MFIS, MFIS-5, and WHOQOL-BREF scoring in hemodialyzed patients.

Questionnaires		MFIS					WHOQOL-BREF			
		MFIS Total Score	5-Item MFIS-5 Score	Physical Subscale	Cognitive Subscale	Psychosocial Subscale	Physical health Domain	Psychological health Domain	Social Relationship Domain	Environment Domain
sex	HD male	40.7 (18.5)	10.4 (4.6)	21.1 (8.9)	15.1 (8.7)	4.5 (2.3)	12.0 (1.6)	12.7 (2.2)	13.2 (3.2)	13.7 (2.3)
	healthy male	22.1 (19.2)	6.0 (4.1)	9.8 (8.6)	9.9 (8.9)	2.4 (2.3)	13.9 (1.3)	15.0 (2.1)	13.3 (3.4)	15.5 (3.2)
	HD vs. healthy male *p*-value	<0.0001	<0.0001	<0.0001	0.0001	<0.0001	<0.0001	<0.0001	N/S	<0.002
	HD female	36.3 (17.9)	9.9 (4.3)	18.6 (8.8)	13.4 (8.2)	4.4 (2.3)	12.0 (2.2)	12.6 (2.2)	13.6 (3.1)	13.6 (2.3)
	healthy female	35.1 (17.4)	8.7 (4.0)	15.8 (8.2)	15.8 (7.8)	3.5 (2.0)	12.6 (1.6)	13.4 (1.9)	13.9 (3.1)	13.2 (2.0)
	HD vs. healthy female *p*-value	N/S	N/S	N/S	N/S	N/S	N/S	N/S	N/S	N/S
age	<median	33.2 (16.9)	8.8 (4.6)	17.5 (8.9)	11.9 (7.3)	3.7 (2.3)	12.2 (1.9)	12.8 (2.2)	13.4 (3.4)	13.5 (2.5)
	>median	45.4 (17.9)	11.6 (4.1)	23.0 (8.1)	7.1 (9.0)	5.2 (2.1)	11.9 (2.0)	12.7 (2.1)	13.7 (2.9)	13.9 (2.2)
	*p*-value	<0.002	<0.002	<0.001	0.003	0.0005	N/S	N/S	N/S	N/S
education	primary	40.8 (21.3)	10.0 (5.2)	20.5 (9.9)	15.8 (9.7)	4.4 (2.6)	11.8 (1.8)	12.7 (2.3)	13.5 (3.5)	13.3 (2.2)
	secondary	38.6 (11.9)	10.8 (3.1)	20.5 (6.9)	13.4 (6.0)	4.7 (1.8)	11.7 (1.8)	12.8 (2.1)	13.7 (2.8)	13.6 (2.0)
	university	35.3 (16.9)	9.9 (4.2)	19.0 (8.5)	11.9 (7.8)	4.4 (1.8)	13.1 (1.5)	13.1 (2.0)	13.9 (2.4)	15.3 (1.9)
	*p*-value	N/S	N/S	N/S	N/S	N/S	0.0003 ^a^ 0.0002 ^b^	N/S	N/S	<0.002 ^a^ 0.0004 ^b^
marital status	married	40.0 (17.1)	10.5 (4.0)	21.0 (7.8)	14.5 (8.6)	4.5 (2.1)	12.2 (2.0)	12.9 (2.1)	13.8 (3.0)	13.9 (2.3)
	single	37.8 (20.7)	9.6 (5.4)	18.8 (10.6)	14.7 (8.5)	4.4 (2.7)	11.8 (1.3)	12.5 (2.3)	13.2 (3.6)	13.3 (2.1)
	*p*-value	N/S	N/S	N/S	N/S	N/S	N/S	N/S	N/S	N/S

MFIS—Modified Fatigue Impact Scale, MFIS-5—abbreviated 5-item version of MFIS, WHOQOL-BREF—World Health Organization Quality of Life questionnaire, HD—hemosialysis, ^a^—primary education versus university, ^b^—secondary education versus university. N/S—*p* > 0.05.

**Table 2:** Dialysis-related factors affecting MFIS, MFIS-5, and WHOQOL-BREF scoring in hemodialyzed patients.

Questionnaires		MFIS					WHOQOL-BREF			
		MFIS Total Score	5-Item MFIS-5 Score	Physical Subscale	Cognitive Subscale	Psychosocial Subscale	Physical health Domain	Psychological health Domain	Social Relationship Domain	Environment Domain
duration of the hemodialysis session	<4 h	37.8 (18.4)	9.7 (4.7)	19.4 (9.8)	13.8 (7.0)	4.6 (2.6)	12.4 (1.9)	13.1 (2.4)	14.7 (3.4)	14.1 (2.0)
	>4 h	32.6 (20.1)	8.2 (5.0)	17.0 (9.9)	12.7 (9.3)	2.9 (2.2)	11.9 (1.7)	12.8 (1.9)	12.8 (2.7)	14.0 (1.9)
	*p*-value	N/S	N/S	N/S	N/S	0.0002	N/S	N/S	<0.002	N/S
Kt/V	<median	38.3 (19.9)	10.4 (4.6)	20.6 (9.6)	13.6 (9.6)	4.1 (2.2)	12.2 (1.6)	13.2 (1.9)	13.9 (2.7)	14.1 (2.0)
	>median	40.8 (16.4)	10.2 (4.4)	20.2 (8.1)	15.7 (7.2)	4.9 (2.3)	11.9 (2.0)	12.3 (2.3)	13.2 (3.5)	13.3 (2.4)
	*p*-value	N/S	N/S	N/S	N/S	N/S	N/S	N/S	N/S	N/S
vascular access	AVF	39.3 (17.9)	10.3 (4.3)	20.4 (8.6)	14.4 (8.5)	4.5 (2.3)	12.0 (1.8)	12.8 (2.2)	13.6 (3.0)	13.7 (2.2)
	CVC	39.7 (22.0)	9.7 (6.2)	19.7 (11.2)	15.6 (8.9)	4.4 (2.5)	12.5 (1.7)	12.4 (1.9)	13.3 (3.7)	13.5 (2.5)
	*p*-value	N/S	N/S	N/S	N/S	N/S	N/S	N/S	N/S	N/S
URR	<median	40.8 (20.7)	10.8 (4.8)	21.4 (10.0)	14.9 (9.9)	4.4 (2.4)	12.0 (1.6)	13.0 (2.1)	13.6 (3.1)	13.8 (2.1)
	>median	38.0 (15.7)	9.6 (4.2)	19.3 (7.5)	14.2 (7.1)	4.5 (2.2)	12.1 (2.0)	12.5 (2.1)	13.5 (3.1)	13.7 (2.3)
	*p*-value	N/S	N/S	N/S	N/S	N/S	N/S	N/S	N/S	N/S

MFIS—Modified Fatigue Impact Scale, MFIS-5—abbreviated 5-item version of MFIS, WHOQOL-BREF—World Health Organization Quality of Life questionnaire, AVF—arteriovenous fistula, CVC—central venous catheter, URR—urea reduction ratio. N/S—*p* > 0.05.

**Table 3:** The demographic characteristics of hemodialyzed patients and healthy controls, and scoring of MFIS and WHOQOL-BREF questionnaires.

			Hemodialyzed Patients	Healthy Controls	
		Scoring Range	n/M [Range] (SD)	n/M [Range] (SD)	*p*-Value
sex	male		79 (68.7%)	76 (67.9%)	N/S
	female		36 (31.3%)	36 (32.1%)	N/S
age			63.7 [31–86] (11.5)	60.6 [30–73] (11.9)	N/S
Modified Fatigue Impact Scale (MFIS)	MFIS (full scale)	0–84	39.3 (18.3)	29.6 (19.1)	<0.0001
	MFIS-5 (5-item version)	0–20	10.2 (4.5)	7.6 (4.2)	<0.0001
	Physical Subscale	0–36	20.3 (8.9)	13.3 (8.7)	<0.0001
	Cognitive Subscale	0–40	14.5 (8.5)	13.3 (8.7)	N/S
	Psychosocial Subscale	0–8	4.5 (2.3)	3.0 (2.2)	<0.0001
World Health Organization Quality of Life questionnaire (WHOQOL-BREF)	Physical health domain	4–20	11.9 (1.7)	13.3 (1.6)	<0.0001
	Psychological health domain	4–20	12.6 (2.1)	14.5 (1.9)	<0.0001
	Social relationship domain	4–20	13.5 (3.1)	13.8 (3.4)	N/S
	Environment domain	4–20	13.8 (2.3)	14.0 (2.5)	N/S

N/S *p* > 0.05.

## Data Availability

The data presented in this study are available on request from the corresponding author. The data are not publicly available due to privacy restrictions.
